# A coupled experimental and statistical approach for an assessment of SARS-CoV-2 infection risk at indoor event locations

**DOI:** 10.1186/s12889-023-16154-0

**Published:** 2023-07-20

**Authors:** Lukas Siebler, Torben Rathje, Maurizio Calandri, Konstantinos Stergiaropoulos, Tjibbe Donker, Bernhard Richter, Claudia Spahn, Manfred Nusseck

**Affiliations:** 1 Institute for Building Energetics, Thermotechnology and Energy Storage (IGTE), University of Stuttgart, Pfaffenwaldring 35, Stuttgart, 70569 Baden-Württemberg Germany; 2 Institute for Infection Prevention and Hospital Epidemiology, University Medical Center Freiburg, Breisacher Straße 115 B, Freiburg, 79106 Baden-Württemberg Germany; 3 Freiburg Institute for Musicians’ Medicine, University of Music Freiburg, University Medical Center Freiburg, Medical Faculty of the Albert-Ludwigs-University Freiburg, Freiburg Center for Research and Teaching in Music, Germany, Elsässer Straße 2m, Freiburg, 79110 Baden-Württemberg Germany

**Keywords:** Infection risk assessment, Simulation tool, Large event venue, Ventilation, Airborne transmission, SARS-CoV-2

## Abstract

Indoor event locations are particularly affected by the SARS-CoV-2 pandemic. At large venues, only incomplete risk assessments exist, whereby no suitable measures can be derived. In this study, a physical and data-driven statistical model for a comprehensive infection risk assessment has been developed. At venues displacement ventilation concepts are often implemented. Here simplified theoretical assumptions fail for the prediction of relevant airflows for airborne transmission processes. Thus, with locally resolving trace gas measurements infection risks are computed more detailed. Coupled with epidemiological data such as incidences, vaccination rates, test sensitivities, and audience characteristics such as masks and age distribution, predictions of new infections (mean), situational R-values (mean), and individual risks on- and off-seat can be achieved for the first time. Using the Stuttgart State Opera as an example, the functioning of the model and its plausibility are tested and a sensitivity analysis is performed with regard to masks and tests. Besides a reference scenario on 2022-11-29, a maximum safety scenario with an obligation of FFP2 masks and rapid antigen tests as well as a minimum safety scenario without masks and tests are investigated. For these scenarios the new infections (mean) are 10.6, 0.25 and 13.0, respectively. The situational R-values (mean) – number of new infections caused by a single infectious person in a certain situation – are 2.75, 0.32 and 3.39, respectively. Besides these results a clustered consideration divided by age, masks and whether infections occur on-seat or off-seat are presented. In conclusion this provides an instrument that can enable policymakers and operators to take appropriate measures to control pandemics despite ongoing mass gathering events.

**Supplementary Information:**

The online version contains supplementary material available at 10.1186/s12889-023-16154-0.

## Highlights

- locally resolved trace gas measurement for substance dispersion and infection risk calculation
- coupling with epidemiological data for on-/off-seat infection risks at an exemplary venue (Stuttgart State Opera)
- model based on Monte Carlo method, including sensitivity analysis regarding masks and tests

**Supplementary Information:**

The online version contains supplementary material available at 10.1186/s12889-023-16154-0.

## Introduction

Restrictions to large gatherings as part of the non-pharmaceutical interventions (NPIs) against the spread of SARS-CoV-2 during the COVID-19 pandemic, meant that indoor event locations respectively venues such as theatres and music halls were forced to restrict or cancel their programs. In many countries, abolishment of the most strict NPIs, which roughly fall under the term lock-downs, was deemed possible under certain conditions, particularly as vaccination rates increased. As such, opening of venues was permitted, but often in combination with mandatory mask-wearing and/or testing before entrance. However, this is done without knowing the actual infection risk at specific venues, making it difficult to discern their contribution to the transmission process under various measures. Consequently, this has an impact on the economic operation of these venues, the visitors’ feeling of safety, and potentially the acceptance of NPI. To enable operators for determining this risk and taking appropriate measures, risk estimation models for these venues are required.

Indoor airborne transmission of viruses is a complex process involving emission by the infected host, dispersion through the room, and inhaling by susceptible individuals [2]. Many of the models for aerosol transmission and infection risk of SARS-CoV-2 in indoor environments [14, 16, 18, 20, 26, 39] are only appropriate for small to medium room sizes, as they often require idealised assumptions (e.g. ideal mixed ventilation) which lose accuracy with the size of the room. In venues, displacement ventilation concepts are often implemented, making virus transmission towards neighbours more challenging to predict. Unobjectionable vertical buoyancy flows are superimposed by critical horizontal flows due to disturbance effects (e.g. cold walls and leaky doors). The estimation becomes even more difficult in large and complex rooms, when relevant parameters are unknown [19, 23, 36].

Moritz, S., Gottschick, C., Horn, J. et al. [21] and Murakami et al. [22] modelled the transmission risks associated with periods of stay off-seat and the associated infection risks. In [21] about 1000 visitors of a pop concert were followed using contact tracking devices, whereby the highest number of primarily short-duration contacts was given at the entrance and during the intermission. With the aid of an agent-based Monte Carlo (MC) method the number of infections caused during the event was estimated. Further approaches related to the SARS-CoV-2 pandemic were conducted in [4, 31, 37, 38]. None of these studies evaluated comprehensively large-scale venues using a combined consideration of access probability of a person being infectious as well as infection risks on- and off-seat (e.g. entrance, intermission, exits).

We argue that such a combined approach is necessary when estimating potential infection risks at venues. Therefore, we developed a physical and data-driven model considering the auditorium and other venues areas to predict the infection risk at large venues using a MC method. Such method is particularly suitable due to the numerous parameters and combinatorial possibilities. We here present a coupled experimental and statistical model with spatial resolution, based on locally resolved trace gas measurements, developed within the framework of a project at the Stuttgart State Theatre (Germany) calculating the airborne infection risk during large events. Depending on the epidemiological data (incidence and reproduction number, vaccination rate and test sensitivity) the access probability of a person being infectious varies. Furthermore, past SARS-CoV-2 infections and recent vaccinations confer immunological protection against infections. All these data influence the overall risk assessment, especially for venues with several hundred people. The model and its implementation in a tool developed in Matlab as well as exemplary results of the Stuttgart State Opera are presented in the following sections. The results provide information on the actual conditions (epidemiological data) and supplementary measures (masks, testing, access) for maintaining the operation of venues. This is important for both operators (maintenance and profitability of events) and policy makers (derivation of NPI and control of infection processes).

## Methodology for overall infection risk assessment

The infection process of a venue is a multi-layered issue. It can be divided into the access probability of a person being infectious, the on-seat and off-seat infection risk. Thereby, the infection activity, described by R-value, incidence and serial interval, among others, determines the number of infectious persons entering the venue. Testing as an entry control has an impact on this quantity, with variables such as type of test (rapid antigen test or Polymerase chain reaction (PCR)) respectively their sensitivity and testing strategy being crucial. Infection risks at venues occur on the one hand within the auditorium (on-seat) and on the other hand in further venue areas (off-seat) such as restrooms, bars and entrances/exits. Visitors are heterogeneous and have different characteristics respectively attributes (e.g. wearing a mask, type of mask and vaccination/recovery status).

In order to take all relevant dependencies into account, we developed an overall model, in which experimental measurements of aerosol transmission are linked with the above-mentioned parameters. A Monte Carlo (MC) method is used, which allows a quasi-random assignment of attributes in order to do justice to the stochastic character of the audience characteristics. In this way, the deterministic character of the calculation is lost, but the tool is more flexible compared to an analytical approach.

### Random generation of a virtual audience

To represent the real audience of a venue the model creates a virtual audience, which is composed of visitors who are assigned attributes (e.g. mask and immunity). Visitors are allocated a specific seat within the auditorium and may encounter other visitors in further venue areas. In order to create the virtual audience, the model requires information on the size of the venue, occupancy density, typical age distributions of the audience, vaccination status of the age groups and epidemiological data such as incidences and reproduction number. The classification of these parameters is presented in Table 1. For each class combination there is a related vaccine effectiveness immune protection due to vaccination $\eta _{ij}$, an incidence $I_{ij}$, and a probability of a person being infectious $p_{ij}$, see Eq. 4. Further parameters used in the model are shown in Tables 3, 4 and 5.

**Table 1:** Classification of visitors and their attributes: probability of a person being infectious ($p_{ij}$), immune protection due to vaccination ($\eta _{ij}$), and incidence ($I_{ij}$)

		$\boldsymbol j\boldsymbol=\mathbf1$			$\boldsymbol j\boldsymbol=\mathbf2$			$\boldsymbol j\boldsymbol=\mathbf3$		
	vaccination	0–17 years			18–59 years			60+ years		
$i= 1$	not vaccinated	$p_{11}$	$\eta _{11}$	$I_{11}$	$p_{12}$	$\eta _{12}$	$I_{12}$	$p_{13}$	$\eta _{13}$	$I_{13}$
$i = 2$	vaccinated > 4 months ago	$p_{21}$	$\eta _{21}$	$I_{21}$	$p_{22}$	$\eta _{22}$	$I_{22}$	$p_{23}$	$\eta _{23}$	$I_{23}$
$i = 3$	vaccinated $\le$ 4 months ago	$p_{31}$	$\eta _{31}$	$I_{31}$	$p_{32}$	$\eta _{32}$	$I_{32}$	$p_{33}$	$\eta _{33}$	$I_{33}$

The attribution of visitors is conducted according to the following procedure. The associated parameter specify ranges. Using the random process, a random number between 0 and 1 is generated. A corresponding attribute is assigned to the visitor depending on the range in which this random number falls. For example: age distribution = 1% for age group 0–17, 47% for age group 18–59 and 52% for age group 60+ [34]. This results in the following ranges (cumulative distribution): 0–0.01, 0.01–0.48, 0.48–1.00. For a random number of 0.42, the visitor is assigned to the second age group. In the following text and figures, this procedure is referred to as random process.

**Fig. 1:**
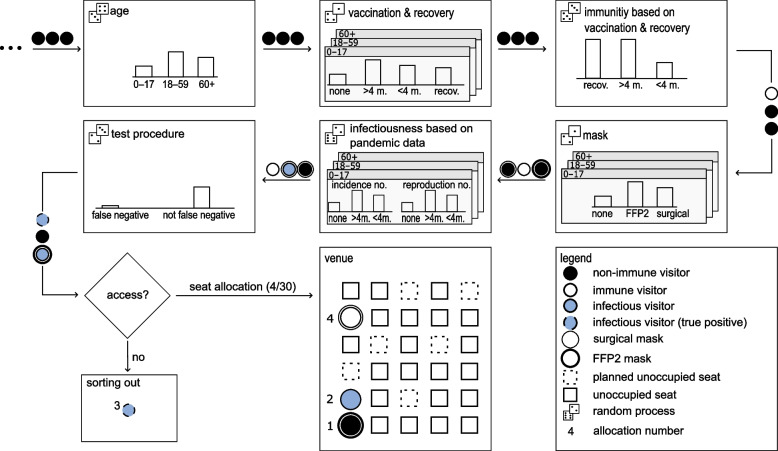
Visitor seat allocation scheme. In each step, visitors are randomly assigned epidemiological properties based on the respective distribution and previously assigned properties

The processes of the model regarding the visitor seat allocation scheme is shown in Fig. 1. This scheme consists of following modules: age, vaccination ($>, \le$ 4 month ago (m.)) & recovery (recov.), mask (none, surgical and filtering face piece 2 (FFP2)), immunity based on pandemic data, infectiousness based on previous data and test procedure. According to the test results, visitors are either sorted out (true positive test) or assigned to seats.

Firstly, visitors are assigned to an age group according to the age distribution. Secondly, the age-dependent vaccination & recovery status is attributed. These attributes determine whether visitors are immune due to their corresponding immune protection in the next step. Afterwards, the presence and type of masks are allocated to the visitors. For the masks, both the filter separation efficiency during inhalation and exhalation are taken into account in order to represent the airborne infection process as accurately as possible [17]. Then, the previously assigned attributes such as age and medical status determine which incidence and effective reproduction number the visitor belongs to. Based on these specific data an estimation of how likely each visitor is currently infectious is carried out. This estimation is based on a forecasted incidence, as reported incidence generally describe the rate of (new) infections per inhabitants at a given time with delay. In the following this mathematical procedure is described in detail.

#### Probability of a person being infectious

We assume that once someone knows that they are infectious they generally stay quarantined or at least they do not try to get access to an event location. Unwittingly infectious persons, both presymptomatic and asymptomatic are therefore assumed to be critical to the within-venue infection process.

For persons who want access to the venue the probability of being infectious is calculated by predicted incidence rates. This forecast can be estimated by the effective reproduction number $R_{\textrm{e}}$ (effective R-value), the serial interval (mean duration between being infected and infecting the next person) $\Delta t_{\textrm{si}}$ and the mean critical duration $\Delta t_{\textrm{crit}}$ between one becomes infectious and the knowledge about it for presymptomatic persons respectively the end of infectiousness for asymptomatic persons. Since the determination of the ratio of asymptomatic persons is challenging, an alternative approach is to use a slightly higher $\Delta t_{\textrm{crit}}$ than suggested by He et al. [11] in order to cover both groups. $R_{\textrm{e}}$ describes how many persons one infectious person infects on average under the current epidemic conditions. For even higher accuracy, reproduction numbers could optionally be further classified by age groups and vaccination/recovery status. The following equation shows the correlation between the aforementioned values assuming unhindered exponential course for a certain group (i,j):

1 $$\begin{aligned} I_{ij,0} \, e^{r\,\Delta t_{\textrm{si}}} = I_{ij,0} \, R_{\textrm{e}} \end{aligned}$$

with $I_{ij,0}, r, \Delta t_{\textrm{si}}, R_{\textrm{e}}$ as initial incidence (with age and vaccination/recovery status related separation), growth rate in 1/d, serial interval (mean duration between being infected and infecting the next person) and effective reproduction number (R-value) respectively. The growth rate (*r*) of the incidence can be derived from Eq. 1:

2 $$\begin{aligned} r = \frac{\ln {R_{\textrm{e}}}}{\Delta t_{\textrm{si}}}. \end{aligned}$$

To estimate the critical ratio of unwittingly infectious persons, who do not know about their infectiousness at a certain day, it is essential to predict the incidence course up to the duration $\Delta t_{\textrm{crit}}$ later. The prediction is based on the following exponential assumption of Eq. 3 with the reproduction number in the exponent:

3 $$\begin{aligned} I_{ij}(t) = I_{ij,0} \, e^{\frac{\ln {R_{\textrm{e}}}}{\Delta t_{\textrm{si}}} \, t}. \end{aligned}$$

The integral of that course over the critical duration describe the probability for a certain person being in that critical period of infectiousness ($\Delta t_{\textrm{crit}}$) without knowing it at a certain time. This is based on the fact that the incidence as a ratio of infected persons to inhabitants already has the character of a probability.

For a certain class combination (i,j) it is calculated as:

4 $$\begin{aligned} p_{ij} = \int \limits _{0}^{\Delta t_{\textrm{crit}}} I_{ij,0} \, e^{\frac{\ln {R_{\textrm{e}}}}{\Delta t_{\textrm{si}}} \, t} \, \textrm{d}t = I_{ij,0} \, \frac{\Delta t_{\textrm{si}}}{\ln R_{\textrm{e}}} \, \left( \, e^{\frac{\ln {R_{\textrm{e}}}}{\Delta t_{\textrm{si}}} \, \Delta t_{\textrm{crit}}} \, -1 \right) \end{aligned}$$

with $p_{ij}$ and $I_{ij,0}$ as probability of a person being infectious of a certain class combination *ij* and the the initial or current incidence of a certain class combination *ij* respectively.

Once the probability of a person being infectious has been calculated, the testing procedure for entrance of the venue is carried out (Fig. 1). For each group, test requirement and sensitivity (depending on the type of test used) is set according to [12, 32] in terms of false negative results. In case of an infectious visitor, the model determines whether this visitor is actually subsequently detected (no. 3 in Fig. 1) or granted access due to a false negative test result, based on the random process described before.

The following seat allocation can now be done sequentially, since the attributes are already randomly assigned. By specifying an occupancy density, seats can be intentionally left empty (planned unoccupied seat) due to venue utilization or NPI. If a visitor is sorted out due to a true positive test result, the reserved seat will not be occupied (unoccupied seat). Hence, it is assumed that the ticket respectively the corresponding seat will not be passed on. As a result, fewer seats may be occupied at the end of the seat allocation process.

### Substance dispersion measurements

For transferring measurement data into airborne infection risks a mathematical approach is required. The Wells-Riley model [15, 27] allows an estimation of the predicted infection risk via aerosols (PIRA). They introduced quanta as a fictitious unit for an amount of inhaled viruses which lead to an infection with a certain probability. As a result the following correlation is announced:

5 $$\begin{aligned} P_{\textrm{I}} = 1 - \textrm{e}^{-D_{\textrm{q}}} \end{aligned}$$

with $P_{\textrm{I}}$ and $D_{\textrm{q}}$ as PIRA and dose of inhaled quanta, respectively.

#### Substance dispersion measurements at individual positions

Siebler et al. [33] presented two experimental methods for substance dispersion with surrogate particles and trace gas suggesting the latter for ventilation with outdoor air exchange. It is based on releasing a certain rate of gas with a mass flow controller. Measuring the neighboring concentrations with a gas analyser enables users the quantification of substance dispersion in general. Assuming that trace gases are dispersed in the same way as relevant virus-bearing aerosols [3], a transfer to infection risk is possible. In order to link trace gas to quanta and to account for mask filtration effects the introduced approach is now adapted to quasi-stationary concentrations (conservative assumption) of trace gases for practicable implementation in the tool:

6 $$\begin{aligned} D_{\textrm{q}}&= (1-\eta _{\textrm{inh}})\,\dot{V}_{\textrm{inh}} \int \limits _{0}^{t_{\textrm{exp}}} c_{\textrm{q}}(t) \, \textrm{d}t \approx (1-\eta _{\textrm{inh}})\,\dot{V}_{\textrm{inh}} \, c_{\textrm{q,steady}}\, t_{\textrm{exp}} \end{aligned}$$

7 $$\begin{aligned}&c_{\textrm{q,steady}} = (1-\eta _{\textrm{exh}}) \, \frac{\dot{q}_{\textrm{out}} \, M_{\textrm{tg}}}{\dot{m}_{\mathrm {out\,tg}}} \, \frac{\rho _{\textrm{air}} }{M_{\textrm{air}}} \, c_{\textrm{tg,steady}} \end{aligned}$$

with $c_{\textrm{q}},c_{\textrm{q,steady}}, t_{\textrm{exp}}, \eta _{\textrm{inh}}, \eta _{\textrm{exh}}, \dot{q}_{\textrm{out}}, M_{\textrm{tg}}, \dot{m}_{\mathrm {out\,tg}}, \rho _{\textrm{air}}, \dot{V}_{\textrm{inh}}, M_{\textrm{air}}, c_{\textrm{tg,steady}}$ as quanta concentration, its quasi steady concentration, exposition time, mask filtration efficiency for inhaling/exhaling, exhaled quanta rate, molar mass of trace gas, mass flow of trace gas (output), density of air, inhalation volume flow, molar mass of air and measured trace gas concentration (steady) respectively. In order to calculate the dose a numerical integration of trace gas concentration is needed. Filling in equation (5) results in the predicted infection risk via aerosols for a certain position [33].

#### Transfer of individual measurement positions on entire venue

**Fig. 2:**
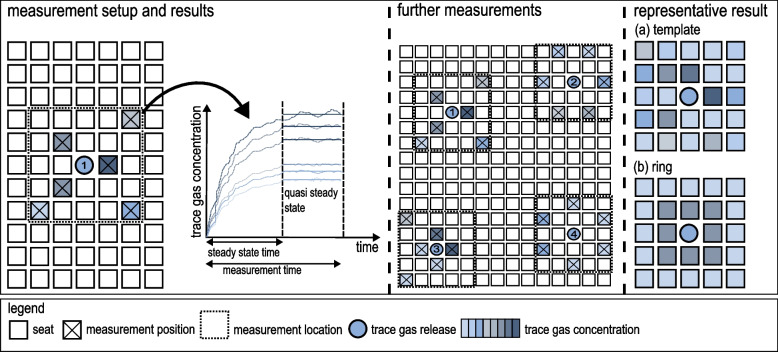
Measurement procedure and transfer into representative result. On certain measurement locations trace gas concentrations are determined after reaching quasi steady state at each measurement position. The results of all locations are averaged either (a) over the exact position relative to the trace gas release or (b) within zones surrounding the release

The previously presented equations can be used to calculate the infection risk of every position. Inside the venue several measurement locations are chosen. Each consists of a single release position of the trace gas and several surrounding measurement positions, terms: see legend of Fig. 2. Assessing the infection risk at venues requires a method that aggregates several results of different measurement locations. At first, these locations have to be defined. Depending on the present ventilation principle (mixing ventilation, displacement ventilation, downward ventilation), the air flow should be analysed on site. In venues, displacement ventilation concepts are commonly applied. These are particularly sensitive to disturbances caused by leaks and opening of windows/doors, downdrafts, etc. [19, 23, 36], which may affect the substance dispersion. Using fog machines, critical areas (e.g. seperated flow regions, low ceiling heights) could be identified.

In the next step, the measurement positions within a certain measurement location have to be defined, see Fig. 2. Often the measurement equipment respectively the quantity of measurement positions are limited. On the one hand, the measurement positions can be quantified by random sampling. Thereby, these positions should be well distributed in case of a small number of measurement locations. If horizontal air flows, which might predominate over the momentum of exhalation, are not known, a 360$^{\circ }$ circumferential arrangement of measurement positions is recommended. On the other hand, the measurement positions can be selected in critical areas subsequent to fog experiments for a conservative assumption. The reference of measurement positions to the trace gas release can either be seat-independent with fixed distances or relative to the seat position (row and column). For the latter, it should be noted that the offset of the rows (no direct front neighbour for a better view to the stage) leads to slightly varying distances. Due to practicable implementation in the model, no offset of the rows is taken into account.

The measurements are performed as long as an equilibrium (quasi steady state) between inflow and outflow of trace gas is reached at each measurement position respectively its control volume. Further measurements take place at other locations. For an assessment of the entire venue, all measurements are aggregated into a single representative measurement result. For this purpose, there are several methods, with two of them being illustrated in Fig. 2: (a) template and (b) ring.

For the former, all results are aggregated in a template. Thereby, averaging or maximum value calculations (conservative assumption) of an identical relative measurement position (e.g. frontal neighbor to the release position) are done. If no result is available for a specific relative measurement position, this value can be interpolated or the maximum concentration value of all measurements can be assumed (conservative assumption).

Another approach is to define zones, e.g. near field (grey area) and far field (bright blue area), see Fig. 2(b). All results inside the respective zone are averaged or a maximum value is determined. Even though the measurement effort might be less elaborate, this averaging could cause a loss of information about specific effects of the air flow (e.g. lateral disturbances). The introduced aggregation procedures according to Fig. 2a or b leads to a representative measurement result, applicable for the entire venue. In the model, the trace gas concentration (steady) and its corresponding mass flow (output) according to Eq. 7 of the representative result, see exemplarily Fig. 5(c), are assigned to the surrounding seats of every infectious person. Here, Eqs. 5, 6 and 7 are evaluated individually in order to determine the infection risk at each seat.

### On-seat infection risk

**Fig. 3:**
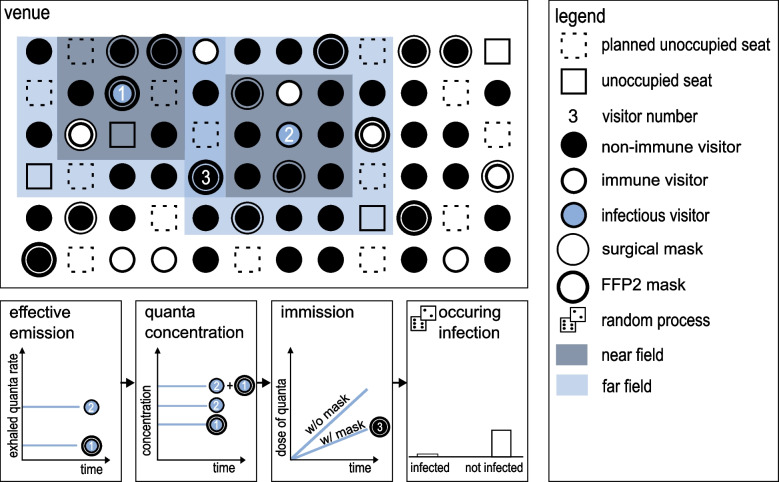
On-seat infection process model. Exemplary Consideration of person No. 3: Infectious persons No. 1 and 2 are emitting a certain quanta rate based on their masking. Based on an overlapping for person No. 3 this results in a accumulated quanta concentration. Due to a FFP2 mask the dose of inhaled quanta is reduced. A Monte Carlo Method uses the resulting infection risk for the decision whether an infection occurs

The individual on-seat infection risk significantly depends on the attributes of the respective visitors (infectious and susceptible). The generation of the virtual audience described in Section “Random generation of a virtual audience” (Fig. 1) and the representative measurement result (Section “Transfer of individual measurement positions on entire venue”, Fig. 2) are therefore merged. As illustrated in Fig. 3, the attributes non-immune, immune, infectious and mask are listed.

the representative result (here exemplarily as ring with corresponding near and far field, see Section “Transfer of individual measurement positions on entire venue” and Fig. 2(b)) is applied. Immunity is determined according to the vaccination/recovery status based on the random process. If an infectious person is generated, the representative measurement result (here exemplarily as ring with corresponding near/far field, see Section “Transfer of individual measurement positions on entire venue”, Fig. 2(b)) is applied. The characteristics of the masks are considered separately in terms of filtration efficiency for exhalation and inhalation. Thus, the emission of an infectious person and the immision of a susceptible person are taken into account accurately.

In case of close distance between several infectious persons, the representative measurement results are evaluated according to a superposition. This effect occurs exemplarily for visitor no. 3, as they are exposed to the emissions of the infectious persons no. 1 and 2. Given that visitor 1 wears an FFP2 mask, their emission is lower than the one of visitor 2. In this case, the quanta concentration according to Eq. 7 results from their superposition. Since visitor 3 also wears an FFP2 mask, the dose of inhaled quanta is reduced (Eq. 6). Finally, the on-seat infection risk $P_{\mathrm {I,on-seat}}$ is calculated according to Eq. 5. This value is used for the random process to determine whether the person becomes infected. The principle is applied to all visitors within the exposition area of the infectious persons.

### Off-seat infection risk

**Fig. 4:**
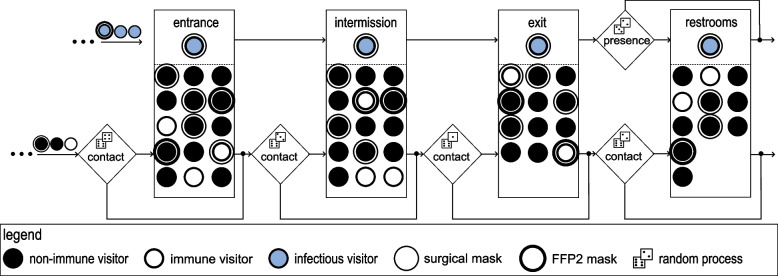
Off-seat infection process model. For each infectious person the tool randomly picks a group of contacts at each venue area, which determines the infection risk for each contact

In addition to the infection risk when sitting in the auditorium (on-seat), there are also further venue areas (off-seat) where visitors could come into contact with each other and become infected. Those places are mainly the entrance hall, the reception hall during the intermission, the exits and optionally the restrooms as illustrated in Fig. 4. A different number of visitors with various exposition times meet here, which makes it rather difficult to determine the potential infection risk in these scenarios via Eq. 5.

Therefore, risks are calculated for each venue area individually and then combined. The average ratio of visitors in contact with each other (ratio of contacts) and the corresponding infection risk without mask was parameterised using values from existing studies [8, 21, 22].

Having generated and attributed the virtual audience (non-immune, immune, infectious, masks, among others), the contacts are considered from the point of view of the infectious persons. The infectious person stays in the entrance hall, the reception hall during the intermission, the exit and possibly in the restroom. Then, according to the ratio of contacts, a random process determines whether a visitor has contact with this infectious person in the respective venue area.

In case of a non-immune visitor, the infection risk is assigned corresponding to the mask filtration efficiency (exhalation, inhalation) of the infectious and susceptible person. For mask wearing, a distinction is made between on-seat and off-seat, resulting in the following combinations: none – none, none – surgical, none – FFP2, surgical – surgical, FFP2 – FFP2 (on-seat – off-seat). Thus, it is assumed that in some cases no mask is worn on-seat and that when walking if necessary a mask is put on or the previously worn mask is kept on.

### Total infection risk

In the end, the individually calculated infection risks of a single visitor in the respective venue areas are combined into the total infection risk of this person according to Eq. 8:

8 $$\begin{aligned} P_{\textrm{I,total}} = 1-\big[\underbrace{(1-P_{\mathrm {I,on-seat}})}_{\begin{array}{c} \mathrm {on-seat} \end{array}} \, \underbrace{(1-P_{\textrm{I,entrance}}) \, (1-P_{\textrm{I,restrooms}}) \, (1-P_{\textrm{I,intermission}}) \, (1-P_{\textrm{I,exit}})}_{\begin{array}{c} \mathrm {off-seat} \end{array}} \big] \end{aligned}$$

where the total infection risk $P_{\textrm{I,total}}$ for a single visitor is calculated from the on-seat and off-seat infection risks in the different venue areas. Finally the random process determines whether this visitor gets infected based on $P_{\textrm{I,total}}$.

## Examplary results of a large venue

###  Measurement results and model parameter

**Fig. 5:**
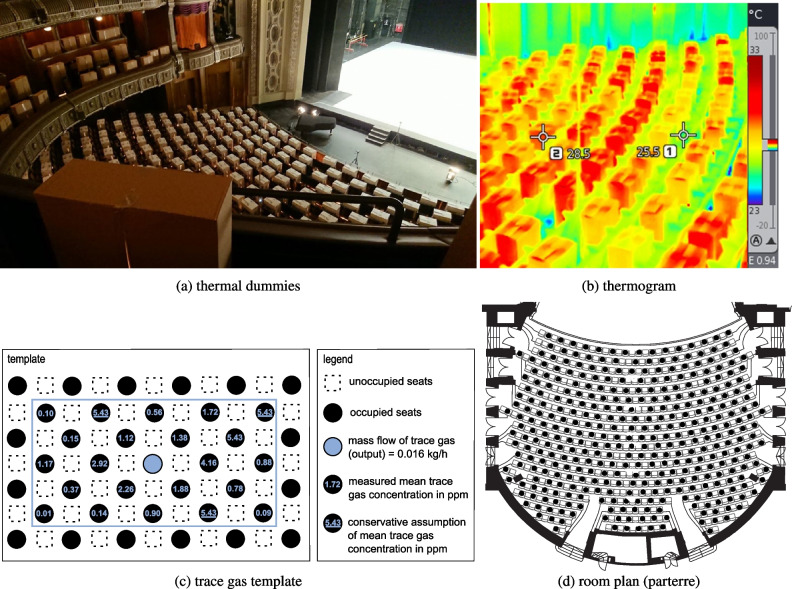
Measurement procedure in Stuttgart State Opera. The placement of (**a**) the thermal dummies, their (**b**) thermogram, including two exemplary points (1: surface temperature of a dummy, 2: between the seats), (**c**) trace gas template, and (**d**) the placement of the thermal dummies on the room plan

Back in 2021, the authors conducted numerous trace gas measurements to assess the airborne infection risk at the Stuttgart State Opera (Germany). Given that an occupancy density of 50% was appropriate for the pandemic situation at the time, half of the seats, 692 of 1404, were equipped with thermal dummies in a chessboard layout, see Fig. 5a, b and d. Furthermore, back then, the assumption was made that only one infectious person would attend the venue, as well as that none of the visitors was protected against infection through vaccination. Due to the fact that the boundary conditions in terms of the infection situation, vaccination protection, testing and the NPI have constantly changed in the course of the pandemic, the current model has been developed to enable the holistic assessment of the infection risk at the venue in a flexible and detailed way.

At the Stuttgart State Opera, a displacement ventilation principle is in operation with an approximate volume flow of 46 000 m^3^ h^-1^. The thermal loads were 55 kW (thermal dummies) resp. 111 kW (lighting). Sulfur hexafluoride (SF$_{\textrm{6}}$) was used as trace gas medium. The devices used included a Bronkhorst F-201CV mass flow controller and a Gasmet DX4015 spectrometer. Further measurement-relevant data can be taken from Table 2.

**Table 2:** Measurement parameters of trace gas experiments

Quantity	Value	Quantity	Value
seats	692/1404	vent. volume flow	ca. 46000 m^3^ h^-1^
floor space	stages: 1170 m^2^	ratio of outside air	100%
	auditorium: 994 m^2^	measurement time	1 h
room volume	stages: 23414 m^3^	trace gas medium	SF_6_
	auditorium: 8149 m^3^	trace gas mass flow	0.016 kg h^-1^
thermal loads	thermal dummies: 55 kW	MFC^a^	Bronkhorst F-201CV
	lighting: 111 kW	FTIR^b^	Gasmet DX4015

^a^ mass flow controller

^b^ Fourier-transform infrared spectroscopy

The representative measurement result (according to Section “Transfer of individual measurement positions on entire venue”, Fig. 2(a), can be found in Fig. 5(c). Values of the trace gas concentration (measured values averaged over the respective measuring position) are given in ppm. For three positions no measured values were available. Therefore, these values (indicated underlined) were conservatively assumed to be the maximum value of all measurement positions.

Model parameters, here subdivided into measurement results, general data, immune protection based on status and contacts and off-seat infection risk are listed in Table 3, and age-dependent parameters are shown in Table 4. The values of the vaccination protections refer to previous studies and are classified into recovered persons [10], vaccinations less than four month ago and more then 4 month ago [24]. The numbers of off-seat encounters are estimated as percentages of the audience size, determined according to the specific venue. They are related to previous findings from other studies [21, 22]. The off-seat infection risks in each venue area are estimated according to their size and their assumed duration of encounters [8]. The values given refer to the infection risk without carrying a mask. The model then determines the reduction of the risk according to the type of the mask of the infectious and susceptible visitor [17].

**Table 3:** Model parameters

Quantity	Value	Quantity	Value
*Measurement results*			
trace gas (SF$_{\textrm{6}}$) mass flow	0.016 kg h$^{-1}$	molar mass of trace gas (SF$_{\textrm{6}}$)	146 g mol$^{-1}$
measurement result principle	‘template’		
*General data*			
exposition time	3 h	filt. efficiency exhal. (surgical)	0.5 [17]
quanta emission rate	114.5 h$^{-1}$ [1, 25]	filt. efficiency exhal. (FFP2)	0.7 [17]
effective reproductive number	1.03 [30]	filt. efficiency inhal. (surgical)	0.5 [17]
prob. false neg. test (PCR)	5% [9]	filt. efficiency inhal. (FFP2)	0.7 [17]
prob. false neg. test (rapid antigen test)	20% [9]	correction factor for incidence	2.0
serial interval	6 d [7]	critical period of infectiousness	2 d [11]
*Immune protection based on status*			
recovered	80% [10]	vaccinated > 4 months ago	30% [24]
vaccinated $\le$ 4 months ago	80% [24]		
*Contacts and off-seat infection risk*			
venue area	ratio of contacts [21, 22]	venue area	inf. risk w/o mask [8]
entrance hall	3%	entrance hall	2%
restrooms	2%	restrooms	3%
intermission	5%	intermission	3%
exits	3%	exits	2%

**Table 4:** Age-dependent model parameters (reference date: 2022-11-29)

	Age groups		
	0–17	18–59	60+
Quantity	Value	Value	Value
*General data*			
age distribution attendees [34]	1%	47%	52%
incidence^a^ [29]	39.4	148.3	127.0
*Vaccination status*			
not vaccinated [28]	71.2%	18.6%	11.1%
vaccinated > 4 months ago [28]	25.8%	72.9%	79.6%
vaccinated $\le$ 4 months ago [28]	3.0%	8.5%	9.3%
*Recovery status*			
recovered since 2022-01-01 [29]	36.9%	41.5%	19.7%
*Proportion of masks*	see Table 5		
*Access* | *test*	see Table 5		

^a^ cases per 7 days per 100k inhabitants

For the age-dependent parameters, the proportion of masks and access/test vary by scenario, see Table 5. The reference scenario represents the status of epidemiological data on 29. November 2022. At that time, there are no access restrictions and testing obligations at the Stuttgart State Opera. The proportions of mask are estimated. A differentiation is made between on-seat and off-seat mask-wearing. It is assumed that a large proportion of visitors do not wear a mask on-seat. If a mask (surgical or FFP2) is worn on the seat, it would be kept on while walking.

**Table 5:** Model settings for scenarios: reference, maximum and minimum safety

	Age groups		
	0–17	18–59	60+
Quantity	Value	Value	Value
**reference scenario**			
$${proportion\;of\;masks}^a$$			
none – none	90%	80%	70%
none – surgical	3%	6%	9%
none – FFP2	3%	6%	9%
surgical – surgical	2%	4%	6%
FFP2 – FFP2	2%	4%	6%
*access* | *test*			
not vaccinated	access: yes | test: none	access: yes | test: none	access: yes | test: none
vaccinated > 4 months ago	access: yes | test: none	access: yes | test: none	access: yes | test: none
vaccinated $\le$ 4 months ago	access: yes | test: none	access: yes | test: none	access: yes | test: none
**maximum safety scenario**			
$${proportion\;of\;masks}^a$$			
FFP2 – FFP2	100%	100%	100%
*access* | *test*			
not vaccinated	access: yes | test: rapid antigen test	access: yes | test: rapid antigen test	access: yes | test: rapid antigen test
vaccinated > 4 months ago	access: yes | test: rapid antigen test	access: yes | test: rapid antigen test	access: yes | test: rapid antigen test
vaccinated $\le$ 4 months ago	access: yes | test: rapid antigen test	access: yes | test: rapid antigen test	access: yes | test: rapid antigen test
**minimum safety scenario**			
$${proportion\;of\;masks}^a$$			
none – none	100%	100%	100%
*access* | *test*			
not vaccinated	access: yes | test: none	access: yes | test: none	access: yes | test: none
vaccinated > 4 months ago	access: yes | test: none	access: yes | test: none	access: yes | test: none
vaccinated $\le$ 4 months ago	access: yes | test: none	access: yes | test: none	access: yes | test: none

^a^ masks: on-seat – off-seat

The maximum safety scenario, on the other hand, requires the wearing of an FFP2 mask (on- and off-seat). Although all groups of visitors (regardless of age and vaccination/recovery status) have access to the venue, a rapid antigen test test is mandatory. In the minimum safety scenario, there are no masks and no tests required, with unrestricted access for all visitors. The results of these three scenarios are presented in the following section.

### Simulation results

For the assessment of the venue, the model calculates the following parameters: new infections (mean), situational *R*-value (mean), average number of infectious persons with access and individual infection risk. In case of new infections and individual infection risk, the results are additionally subdivided according to age group and mask combination. Analogous to the *R*-value, the situational *R*-value indicates the number of infected persons per infectious person and is thereby related to the venue. The individual infection risk relates to the attributes age and mask. Model parameters and the description of the scenarios are described in the previous Section “Measurement results and model parameter”.

**Fig. 6:**
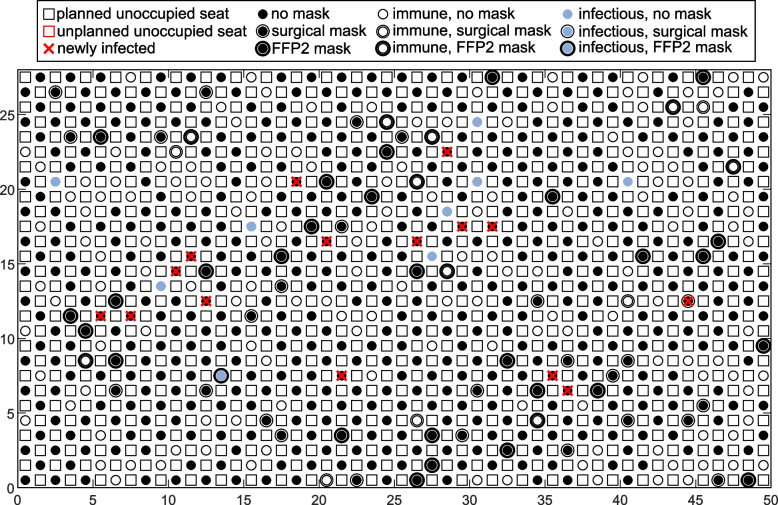
Top view of virtual audience, a single example result of a simulation loop using the reference scenario

A simulation run of a scenario consists of several simulation loops (here: 10000). Each loop consists of all of the above described steps, from generating the virtual audience to determining the new infections based on the total infection risk. Each single loop results in a unique outcome of the infection process. One of these results is illustrated in Fig. 6. Here, a simulation loop was deliberately chosen, in which both an above-average number of infectious persons (9 instead of a mean of 3.8) are present, resulting in disproportionate infections (15 instead of 10.5). Therefore, the relevant facts can be explained based on this figure. The virtual audience with 50 columns (x-axis) and 28 rows (y-axis) 700 visitors are located in a chessboard layout. Since there is no test procedure in the reference case, there are no unplanned unoccupied seats. 9 infectious persons have access to the opera. Two types of infection process (on-seat, off-seat) may be deduced. At 36, 8 and 37, 7 (x,y) are two newly infected persons, without contact to an infectious person on-seat. Therefore, the two visitors must have been infected off-seat. For another example, it can be shown that the infection process might be driven by the on-seat infection risk. There is one infected person at 10, 14 (x,y) with 5 newly infected persons within the zone of the trace gas template. The infection risk is higher due to the mask combination (infectious: none, susceptible: none). Furthermore, at position 12, 16 (x,y) there is a superposition (on-seat) with another infectious person at 16, 18 (x,y).

Running 10000 loops and averaging these values leads to the simulation results of the scenario, as illustrated in Table 6. The simulation run of the reference scenario shows that on average 3.85 infectious persons have access to the venue, with 10.5 persons getting newly infected, which corresponds to a situational R-value (mean) of 2.73. The maximum safety scenario (requirement of FFP2 mask and rapid antigen test) results in 0.25 new infections (mean), 0.75 average infectious persons and a situational R-value (mean) of 0.33. The significant reduction of these parameters shows the effectiveness of the measures taken. In contrast, the minimum safety scenario (no masks/tests) leads to 3.85 infectious persons, 12.9 infected persons and a situational R-value (mean) of 3.35. Compared to the reference scenario, the number of infectious persons is similar (no tests), but the situational R-value is significantly higher, which shows the influence of partial mask wearing.

**Table 6:** Simulation results for different scenarios, showing the mean number of new infections caused within the venue, as well as the mean number of infectious persons that had access to the venue, despite potential testing strategies in place

Scenario	New infections	Infectious persons with access	Situational R-value
Reference	10.6 (10 | 6–14 | 0–24)	3.86 (4 | 2–5 | 1–8)	2.75 (2.67 | 2.0–3.3 | 1.0–5.0)
maximum safety	0.25 (0 | 0–0 | 0–2)	0.75 (1 | 0–1 | 0–3)	0.32 (0.00 | 0.0–0.5 | 0.0–2.0)
minimum safety	13.0 (12 | 8–18 | 1–29)	3.86 (4 | 2–5 | 1–8)	3.39 (3.33 | 2.8–4.0 | 1.5–5.7)

values are given as: mean (median | interquartile range | 95% confidence interval)

**Fig. 7:**
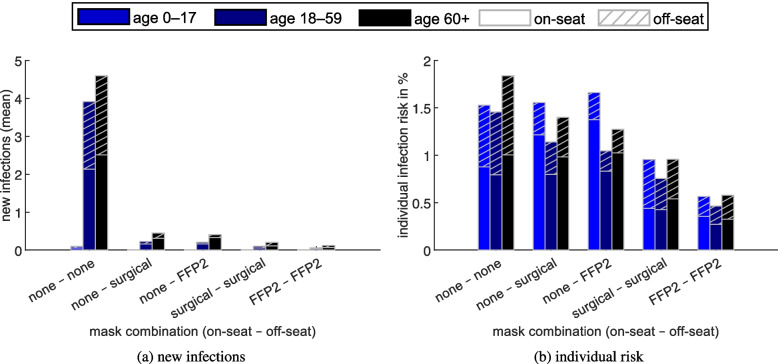
(**a**) New infections (mean) and (**b**) individual risk for different mask combinations (between on-seat and off-seat) in the reference scenario

The influence of mask wearing on new infections (mean) and individual infection risk is shown in Fig. 7 based on the reference scenario. The variables are given as a function of the age group and the mask combination (on-seat – off-seat). Furthermore, each stacked bar is divided into the infection occurring venue areas (either on- or off-seat). It is apparent that the highest number of new infections (Fig. 7(a)) occur in the mask combination none–none due to the fact that they constitute the majority of the audience. The values of the age group 0–17 are only reliable with a very high number of simulation loops given the low proportion of visitors.

Figure 7(b) shows the individual infection risk depending on the age group and the mask combination. The key aspect here is that masks have a significant impact on the individual infection risk, both on- and off-seat. For the two age groups 18–59 and 60+, which together represent nearly all visitors, almost identical on-seat infection risks can be identified for the three mask combinations without masks on-seat. Additionally, off-seat infection risks are similar for the same masks worn off-seat (compare mask combination groups 2 and 4 resp. 3 and 5). This demonstrates the robustness of the simulation model. In the youngest age group 0–17, on the other hand, the patterns described can only be recognised to a limited extent, since the sum of all mask combination groups of this age represents only 1% of the visitors. Therefore, 10000 simulation loops are not sufficient for robust results for this specific age group. In order to better quantify this phenomenon, a plot illustrating the progress of infections per simulation loop is shown in Fig. 9 (appendix).

**Fig. 8:**
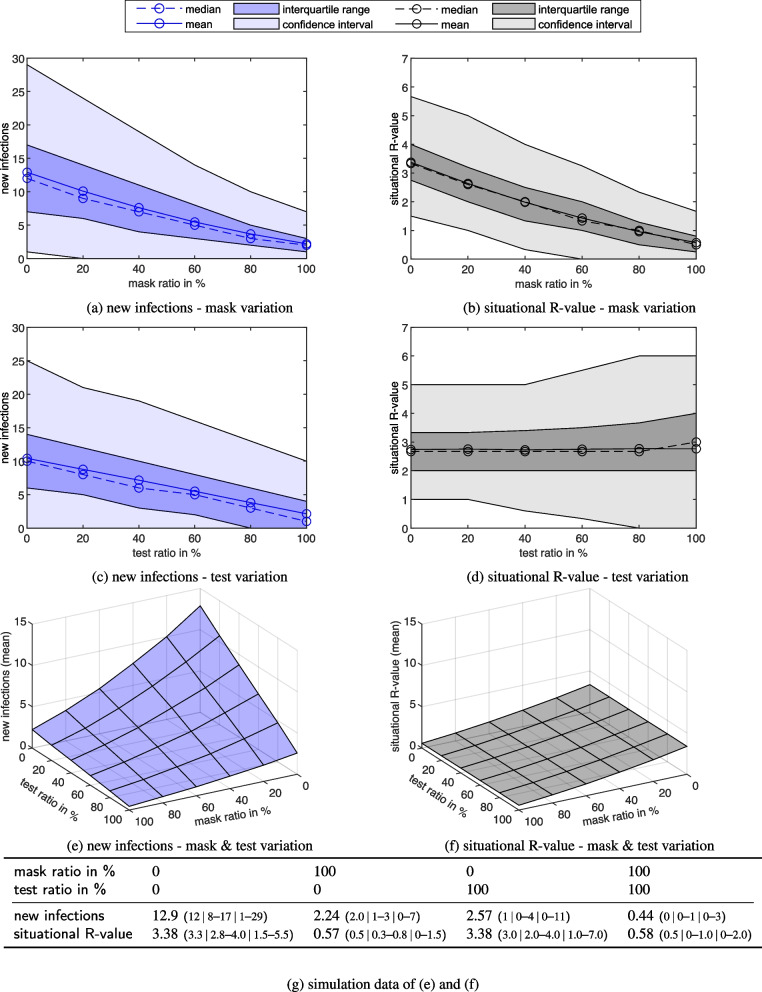
Sensitivity analysis. New infections dependent on mask ratio (**a**), test ratio (**c**) and the compound of both ratios (**e**). On the right side analogously the corresponding diagrams for the situational R-value (**b**), (**d**), (**f**). In the Table (**g**), the simulation outcome values are shown for specific combinations values are given as: mean (median | interquartile range | 95% confidence interval)

As a sensitivity analysis, the ratios of masks and tests were varied based on the reference scenario (Fig. 8). The mask ratio describes the proportion of persons wearing a mask, while the test ratio denotes the proportion of persons who are rapid antigen-tested. The following subfigures illustrate the mean (solid line), the median (dashed line), the interquartile range (dark blue/grey area) and the 95% confidence interval, defined as the 2.5% to 97.5% range of the simulation results (light blue/grey area). Figure 8a and b represent solely the variation of the mask ratio, where, analogous to the reference scenario, no visitor is tested. New infections (mean) range from 2.23 to 12.8, while the situational R-value (mean) ranges from 0.59 to 3.37, both decline linearly with increasing mask ratio. The effectiveness of this measure can be quantified at a mean function gradient of -0.11%$^{-1}$ for the mean value of new infections.

New infections (mean) vary between in a range of 2.04–10.5 (Fig. 8(c)) declining linearly with an increasing test ratio, while the mask ratio remains constant based on the age-dependent values of the reference scenario. With a mean function gradient of -0.08%$^{-1}$ the effectiveness of this measure turns out only slightly smaller for the unchanged remaining parameters of the reference scenario. The ratio of rapid antigen tests performed has no influence on the situational R-value, which stays constant at approximately 2.7 (Fig. 8(d)).

It is worth emphasising that with an increasing mask ratio, both the interquartile range and the confidence interval become narrower in absolute terms, but retain approximately their size in relative terms (see Fig. 8(a)). Similar but reduced effects can be seen for the situational R-value, since the new infections are divided by infectious persons with access and thus a lower variance is to be expected (see Fig. 8(b)). In comparison to masks, with the variation of tests, the interquartile range respectively confidence interval increase in relative terms with higher test ratios for new infections (Fig. 8(c)), and even in absolute terms for the situational R-value (Fig. 8(d)). This could be related to the additional probability of false negative tests, which can lead to a higher variance as the test ratio increases. When mask ratios are varied this effect does not occur, since no tests are performed there.

When considering variation of both parameters (mask and test ratio), we observed (Fig. 8(e)) that for each mask ratio held constant the partial derivative of the new infections after the test ratio turns out to be slightly lower than the partial derivative after the mask ratio for the same test ratios held constant (see also Fig. 8(g)). This result can be interpreted on the basis of the input data: The effect of rapid antigen testing, with a probability of 80% for true positive tests, also provides an approximate reduction in new infections of this magnitude if every visitor is tested, since correspondingly fewer infectious persons gain access. In the case of masks, on the other hand, with an equal ratio of FFP2 and surgical masks within the mask proportion of all visitors, these can provide a hypothetical (deterministically calculated) effect of even slightly below 84% due to mask filtration efficiencies (both during inhalation and exhalation).

While the situational R-value (mean) ranges between 0.59 and 3.37, it is both striking and understandable that the ratio of rapid antigen tests performed has no influence on the situational R-value (Fig. 8(f)). Testing merely ensures that there are fewer infectious persons in the venue. It does not influence the actual infection process neither on- nor off-seat. Here, on statistical average, each infectious person infects an invariant number of persons despite their lower proportion. The effectiveness of the masks on the situational R-value can still be seen with virtually the same gradient as in Fig. 8(e).

## Discussion

The here presented model allows users, such as event operators, to evaluate specific measures such as mask-wearing or testing procedures, by estimating the individual and general infection risk at large indoor venues. The methodology is based on a coupling of experimental and statistical methods. On the one hand, measurement data are recorded and, on the other hand, epidemiological data are analysed. This results in various sources of error, which include measurement uncertainties or challenges in evaluating statistical data such as the age-dependent incidence or the quanta emission rate. Error propagation is difficult to quantify. Therefore, an elaborate experimental design and good monitoring of epidemiological data are basic prerequisites for the application of the model. To test its function and show the extensive simulation data it can generate, the model was parameterised with data from the Stuttgart State Opera as an example.

Regarding the trace gas measurements, its data is averaged and the concentrations in the quasi steady state condition are considered. The non-steady state conditions occurring during operation of the ventilation system or during intermission are thus not covered. Furthermore, it must be examined whether the same boundary conditions (e.g. identical volume flows) are present in all measured areas before averaging. Often, large venues consist of complex geometries, whereby different tiers, loges and the pit have to be considered. The question is what weighting should be applied when averaging the individual measurement locations to an aggregated value for the entire venue. As a criterion, the person-related volume flow of the respective area could be used. Alternatively, several seating areas can be subdivided according to available measurement data respectively trace gas concentrations and modelled separately. However, in the current model, all seats are projected into a two-dimensional area consisting of rows and columns. As a result, on-seat infections are modelled, which would not occur in reality due to the spatial separation (e.g. loge and pit). However, for off-seat modelling it is important that there is a connection between the different locations. The implementation of such features in the present model is very laborious, hence this has been omitted. Besides, this represents a conservative assumption.

**Fig. 9:**
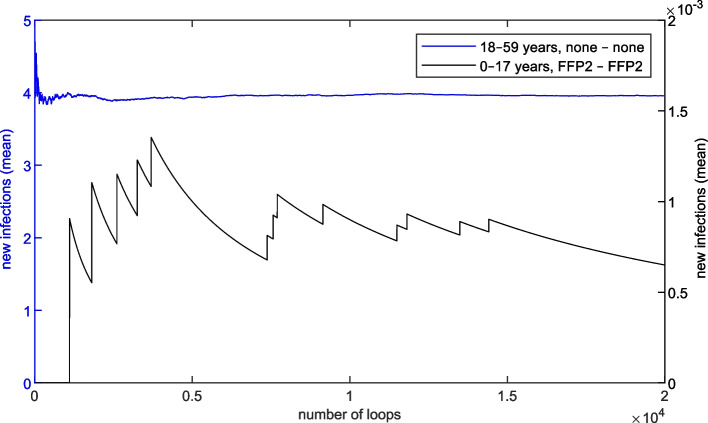
Progress of new infections (mean) dependent on simulation loops, shown for the age group 0–17 with FFP2 masks (right axis) and the age group 18-59 without masks (left axis)

With respect to the model, it must be questioned whether all relevant input parameters were considered. While the derivation of infectious persons through epidemiological data can be assumed as reliable, the attribution of the virtual audience needs to be analysed. Based on the findings of past and current pandemics, the attributes mask, vaccination and test are crucial and the subdivision into age groups, also based on epidemiological data, is a common approach. However, care should be taken to continually consider the incorporation of further classifications and attributes. For further subdivisions, it must also be taken into account that weakly represented groups can postpone the convergence of the simulation results. To ensure their reliability, more simulation loops are required and the effort increases. In Fig. 9 the new infections (mean) depending on the simulation loops for the represented groups with minimum and maximum proportion in the audience of the reference scenario are illustrated. For the most represented age group 18-59 and the mask combination none - none (on-seat - off-seat), no significant change in the value can be seen after only a few thousand simulation loops. For the weakest represented age group 0-17 and mask combination FFP2 - FFP2, on the other hand, this condition is not even recognisable after 20000 simulation loops. It can therefore be deduced that results such as those shown in Fig. 7(a) and (b), especially for the age groups 0-17, are only reliable for much more simulation loops. For very small proportions of groups, however, a compromise between accuracy and computational effort is possible.

Concerning the results, the following can be concluded. The evaluation of the strategy of masks and testing and which measures are to be preferred is contextual. While masks have an impact on both infectious and susceptible persons (exhalation, inhalation) and the situational R-value, the impact of testing is limited to sort out infectious persons without an impact on the situational R-value. However, in case of a high incidence scenario, testing could be preferred and be a more effective measure.

Surprisingly, the new infections (mean) in the age group 60+ were higher than those in the group 18–59. There are two possible reasons for this result. Firstly, the proportion of visitors from the age group 60+ is slightly higher than the others, while their incidence is slightly lower. This makes it more likely that a susceptible 60+ person sits next to an infectious person of age group 18–59 than vice versa. Secondly, the proportion of recently recovered persons in the age group 60+ is lower, which translates into a lower protection through immunity in this group. This lower proportion of past infections in this group is likely caused by a lower number of contacts they have in the general population.

The results from the sensitivity analysis, varying proportions of mask wearing and testing before entrance, are not surprising from the qualitative point of view, but they provide further confirmation of the tool function. With an increasing proportion of tests as well as masks, the new infections decrease. The combination of both measures provides a minimum in this case. The situational R-value, on the other hand, can only be controlled by the proportion of masks, since the number of new infections per infectious person remain is not affected by the entrance testing, which only controls the number of infectious persons entering the venue.

However, the quantitative findings of the parameter variation are actually valuable for both operators and decision-makers. They can use them, for example, to adapt measure strategies precisely to a value of new infections or a situational R-value that is acceptable to them or to the current policy. As such, the influence of ongoing major events on the development of pandemics and epidemics could be actively controlled, instead of merely observed afterwards.

### Limitations

Despite the model being based on a thoroughly parameterised complex and spatially resolved model, with included measurement data for substance dispersion and extensive input options, some limitations need to be addressed. According to the underlying methodology, the movement of visitors is not taken into account and a fixed seat is assumed. Here, temporally and spatially resolved measurements of substance dispersion in the auditorium and other venue areas, RFID-tag contact measurements, as well as simulation of audience movement, could increase accuracy. Otherwise, the model cannot be applied to open-air events or special events where movement is predominant, such as clubs or ballrooms.

Limitations should also be pointed out in the area of the testing process. No false positive tests are considered. These test results could theoretically occur and would result in a healthy -susceptible- person not entering the venue, thus reducing the potential number of newly infected persons. Although, strictly speaking, exclusion of false positive tests would skew the results towards a smaller effect of testing, the total effect is likely negligible because of the relatively high specificity of the tests. Furthermore, the model assumes the wearing of masks with constant mask filtration efficiencies. No maskfitting [5] and no wearout is taken into account, which can significantly affect the mask protection.

The focus of modelling is on the visitors. The artists and especially their instrument related infectious issues [6, 35] are not considered. Under certain circumstances, the staff (for example ticket inspectors or bartenders) can also have a considerable influence on the infection event. However, this represents special cases and is therefore not included for the infection risk assessment of the venue. A good safety and hygiene concept involving the venue’s own staff should be a matter of course.

Moreover, the model assumes that the attributes of the audience (mask wearing, vaccinations status, etc.) are randomly distributed. In reality, people attending an event together would likely have the same attributes and sit together. This clustering of attributes may affect the infection risks. The direction of the clustering effect is hard to predict.

Since the trace gas measurement values are only valid for a measured operating condition and a given occupancy density, a variation of the volume flows or seat occupancy/density cannot be taken into account in the model without further ado. Therefore, the NPI regarding the capacity utilisation of the venue and the mode of operation of the ventilation system must be weighed up from the outset.

In the seating area, only airborne transmission by aerosols was considered. It was assumed that droplet transmission could be neglected in the auditorium because droplets spread mainly when people talk and usually there is no speaking in the audience during the performance. This has also been suspected in other studies [13]. During the intermission, there are conversations at short distances (< 1.5 m) and therefore possible droplet transmissions. This has been considered by the risk anticipation for each venue area, taking into account the numbers of contacts to other visitors and the contact duration regarding findings of other studies [21, 22]. Furthermore, virus transmission via surfaces was also neglected in accordance to Huang et al. [13], since in classical event locations there are only few contact surfaces, except for the seat and in the facilities. Another reason was that the transmission by contaminated surfaces was found to be a thousand times lower than in aerosol transmission [40].

Testing the plausibility of the overall model could already be done on the basis of the simulation results themselves, mainly on a relative and qualitative level. The plausibility of some partial aspects such as the off-seat infection process have already been tested in other studies [8, 21, 22]. For a quantitative plausibility check of the overall results, however, we recommend further measures as an outlook. Based on a specific venue such as the Stuttgart State Opera, seat tracking of each visitor and reporting of subsequent infections could enable such a process. However, it requires an audience that is not bothered by reduced data protection.

Furthermore, sufficient data on SARS-CoV-2 transmission within comparable venues lacks to properly validate the model results. It is therefore difficult to know how accurate the estimated risks compare to actual outcomes of attendance to large cultural events during epidemics. However, through the considerable effort taken in constructing the model, as well as estimating the used parameters, the model should approximate the risks relatively well, in particular when comparing the different combinations of interventions.

## Conclusion

The presented model fulfils the objective of a comprehensive assessment of the infection risk for venues. Standard tools rely on a wide range of assumptions and simplifications. In comparison, our model based on measurements of substance dispersion provides more valuable results. These are particularly important because the consequences of decisions made by policy makers are very high. The results were tested for plausibility and the robustness of the model was successfully demonstrated. A variety of influencing parameters such as epidemiological data, including R-value and incidence, as well as attributes of individual visitors, such as mask and immune protection, are taken into account. It allows for deriving non-pharmaceutical interventions and actively managing the infection processes. Therefore, the model can be successfully used for the purpose of providing policy makers and venue operators a basis for decision making in complex pandemic situations.

## Supplementary Information

**Additional file 1.**

## Data Availability

The datasets used and/or analysed during the current study are available from the corresponding author on reasonable request.
