# Supplementary material for: A coupled experimental and statistical approach for an assessment of SARS-CoV-2 infection risk at indoor event locations
Source: BMC Public Health. 2023 Jul 20;23:1394. doi: 10.1186/s12889-023-16154-0 (PMC10357618; doi:10.1186/s12889-023-16154-0)
Supplement: Supplementary file 1 — Additional file 1. [file 12889_2023_16154_MOESM1_ESM.pdf]

A. Appendix

A.1. Distribution of quanta emission rates

Regarding user-friendliness, we decided to query only a mean value of the quanta emission rate. Finding scientifically reliable values already proves to be very complex and the impact is expected to be negligible. In order to justify this decision, this impact is additionally verified by simulation runs with different distribution curves (fixed value, normal distribution and log-normal distribution) of the quanta emission rate. For the distribution curves (Fig. 10(a) and Fig. 10(b)), the Monte Carlo method decides which emission rate an infectious person emits according to the distribution curve. For the largest group of visitors (age group 18–59 with no mask – no mask (on-seat – off-seat), the mean new infections per simulation loop are therefore calculated and plotted over 30,000 simulation loops (see Fig. 10(c)).

The final values of these curves show only marginal deviations. To explain this effect, the Poisson distribution of the Wells-Riley approach is examined in more detail. For this purpose, a normal distribution of the inhaled quantum dose is formed around the mean value 1. Using 100,000 Monte Carlo loops, the resulting infection risks are now determined via the Wells-Riley approach and then accumulated back into equidistant classes. If the sum of all bars is then normalized to 1, the result is a bar curve superimposed on randomness, which is reminiscent of a log-normal distribution. This transfer is shown in Fig. 11.

If the mean value of the quanta dose normal distribution of 1 would be transferred directly, this would result in the solid black line. However in this new distribution, the numerically determined mean value (dash-dotted black line) is slightly higher. The deviation of both values is of similar magnitude than the final values in Fig. 10(c), which explains this effect. For the sake of simplicity, the standard user should not be faced with the choice of finding parameters of distribution curves.

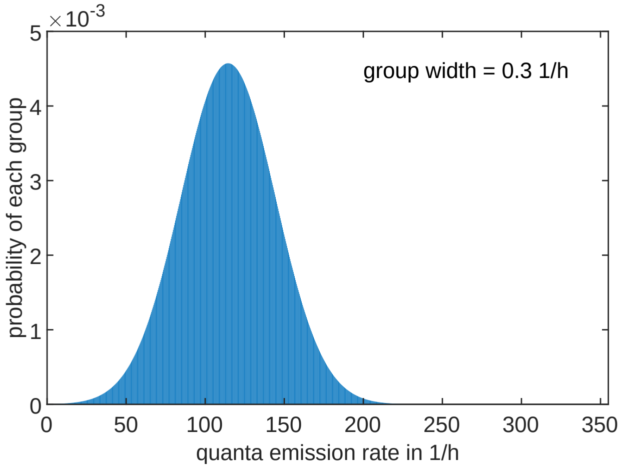

(a) normal distribution of quanta emission rate $\mu = 114.5, \sigma = 30$

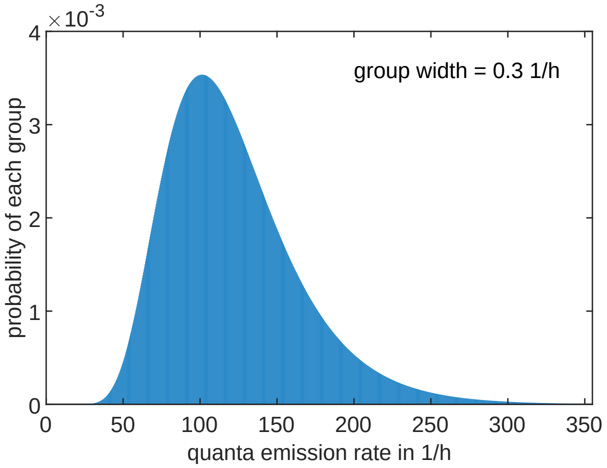

(b) log-normal distribution of quanta emission rate $\mu = \ln(114.5), \sigma = 0.35$

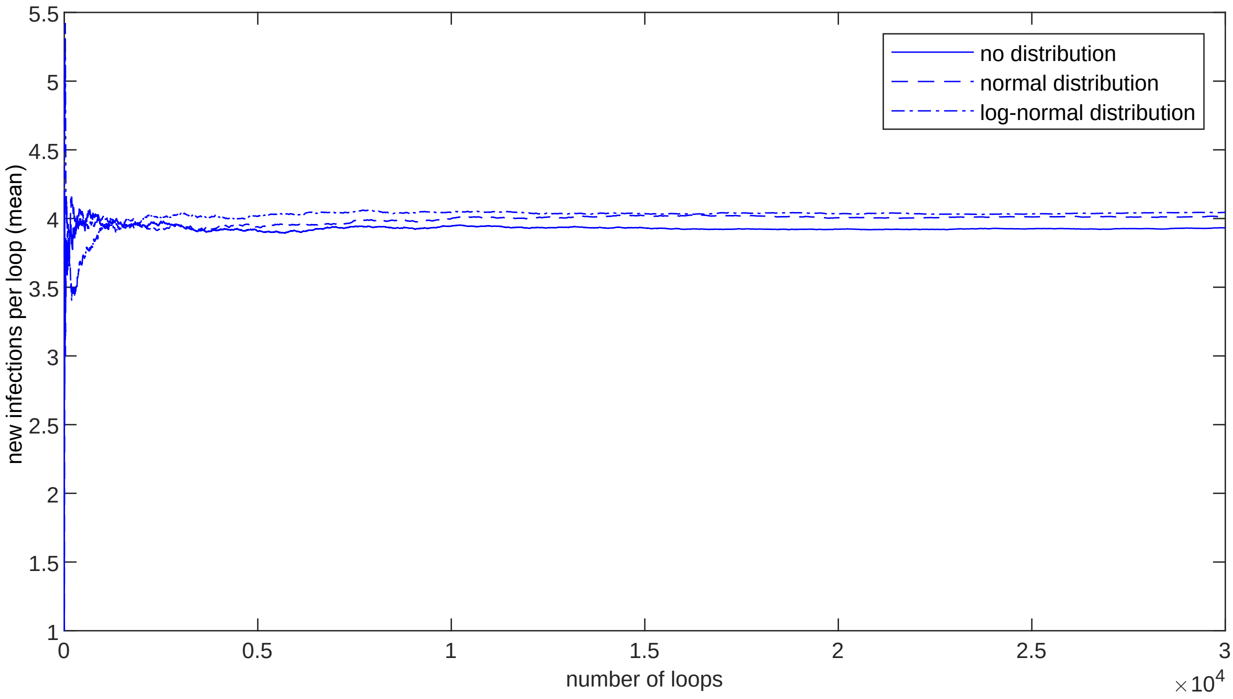

(c) new infections (mean) per loop (age 18–59 & no mask – no mask)

Figure 10: Comparing different input distributions for quanta emission rates. Using a fixed mean, (a) a normal distribution, and (b) a log-normal distribution. The resulting (c) new infections (mean) as a function of the number of simulations loops.

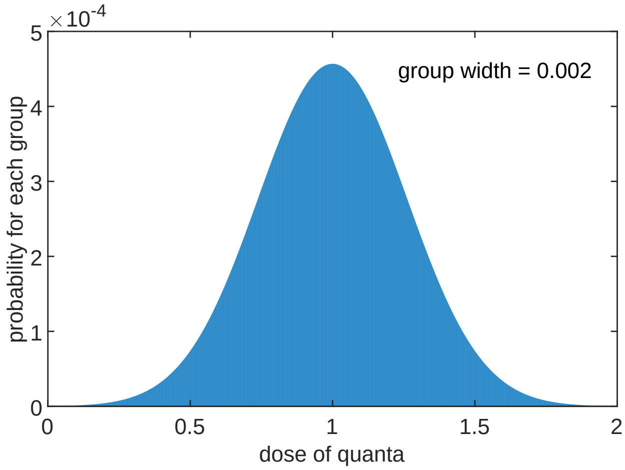

(a) normal distribution of the quanta dose

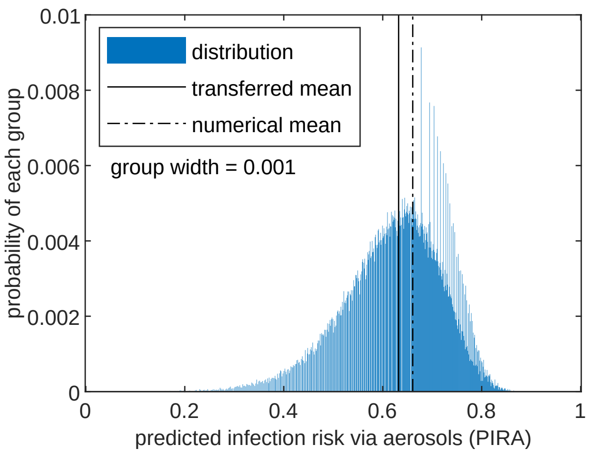

(b) resulting distribution of PIRA

Figure 11: Transfer of (a) a normal distribution of the quanta dose into (b) a PIRA distribution via Monte Carlo method. The transferred mean of the normal distribution (solid line) is slightly lower than the mean of the PIRA distribution (dash-dotted line).

A.2. Topview of minimum and maximum safety scenario

Fig. 12 shows further exemplary topviews of a simulation run for both the minimum safety scenario and the maximum safety scenario. In the minimum safety scenario (Fig. 12(a)), it can be seen that no masks are worn and that, due to missing test requirements, a similar number of infectious persons are present compared to the reference case. In the maximum safety scenario (Fig. 12(a)), on the other hand, only 2 infectious persons have access to the venue since the rapid antigen test result denies access to 4 others (4 unplanned unoccupied seats). Due to the FFP2 mask requirement, no new infections occur despite the presence of two infectious persons in this exemplary case.

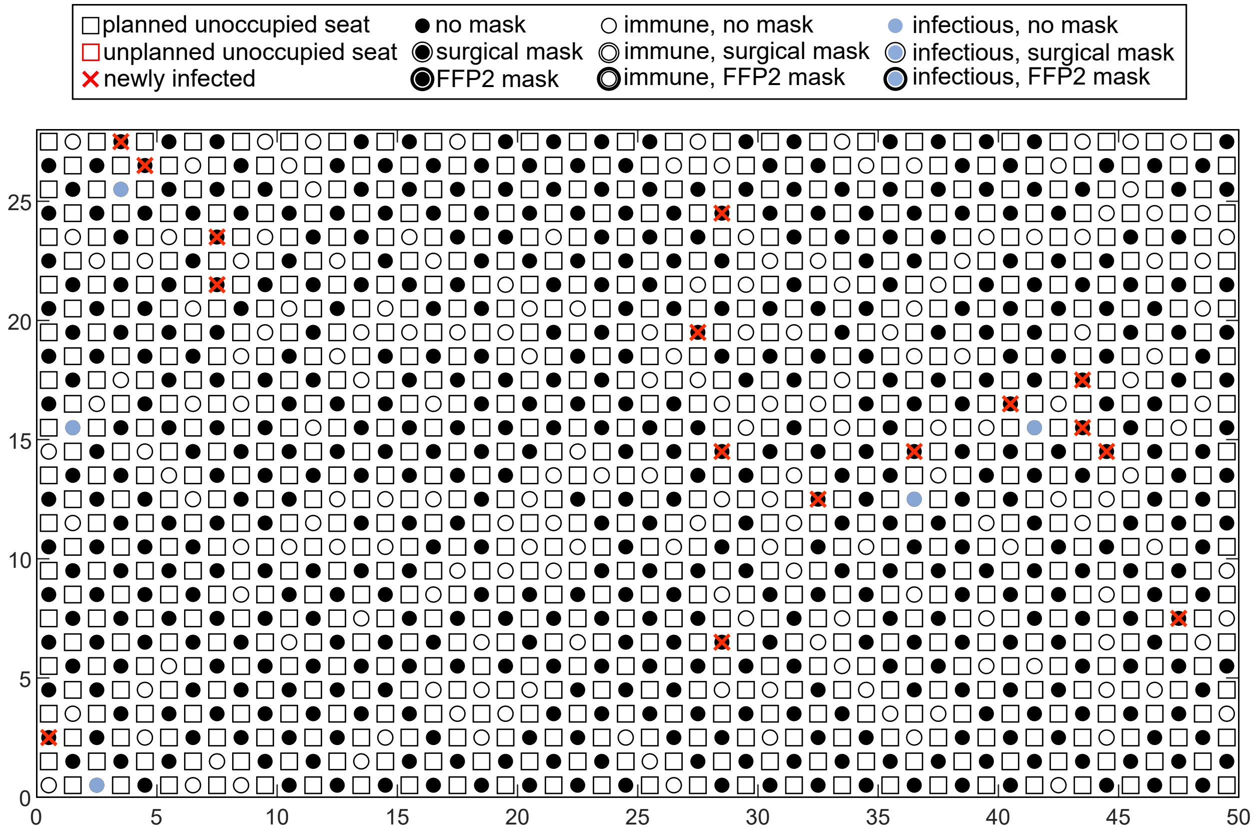

(a) minimum safety scenario

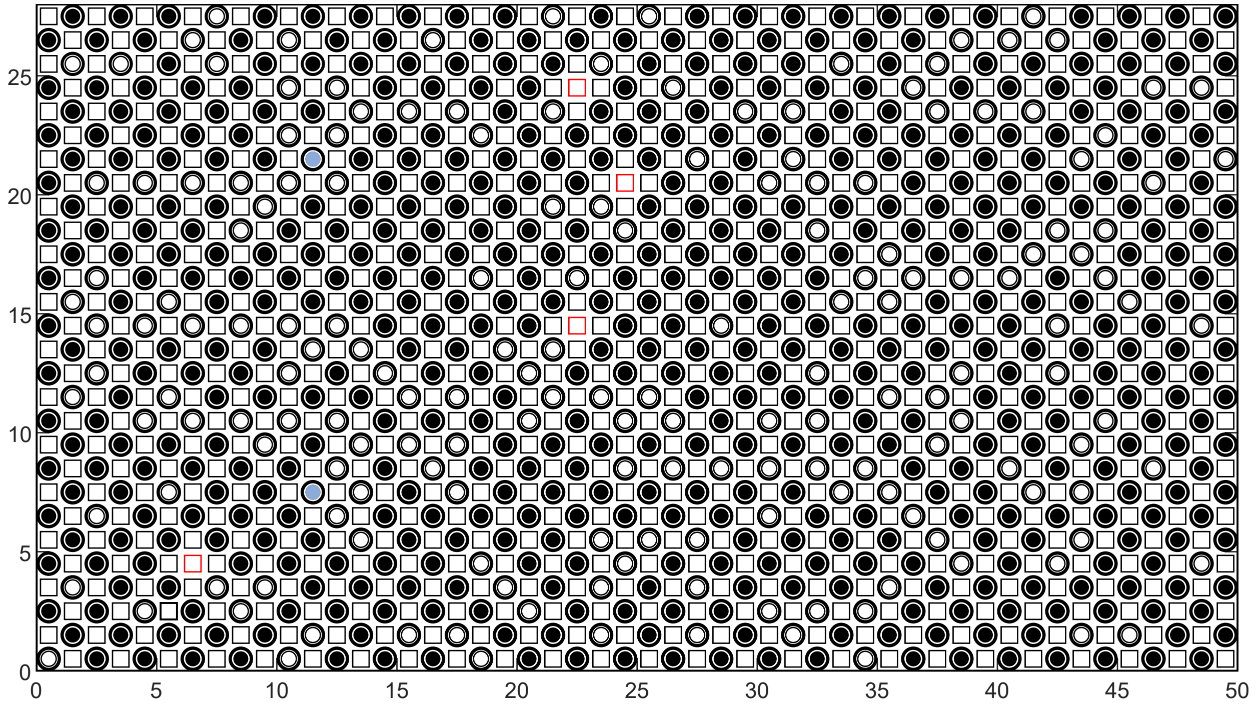

(b) maximum safety scenario

Figure 12: Topview of virtual audience, simulation loop of (a) minimum and (b) maximum safety scenario
